# Cinnamaldehyde is a biologically active compound for the disinfection of removable denture: blinded randomized crossover clinical study

**DOI:** 10.1186/s12903-020-01212-5

**Published:** 2020-08-17

**Authors:** Marco Antônio Lavorato de Almeida, André Ulisses Dantas Batista, Maria Rejane Cruz de Araújo, Vanessa Fabiana Dei Santi de Almeida, Paulo Rogério Ferreti Bonan, Danielle Nóbrega Alves, Tereza Karla Vieira Lopes da Costa, Diego Figueiredo Nóbrega, Ricardo Dias de Castro

**Affiliations:** Faculty of Dentistry, Department of Clinical and Social Dentistry, Federal University of Paraíba, Campus I, João Pessoa, PB 58051-970 Brazil

## Abstract

**Background:**

Fungal infections associated with the use of dentures, like denture stomatitis, are difficult to prevent and treat. This in situ study aimed to investigate the efficacy of cinnamaldehyde for the disinfection of complete removable dentures, and the effect on the physical and mechanical properties (Vickers microhardness, color, and surface roughness) of the acrylic resin.

**Methods:**

Acrylic resin disks were inserted into the dentures of a probabilistic sample of 33 complete denture users, that used cinnamaldehyde (27 μg/mL) and 0.5% sodium hypochlorite solutions in a 20 min/7-days protocol of dentures immersion in each solution, with a wash-out period of 7 days, to constitute a crossover-study. The disks were analyzed before and after the immersion, for the presence of microorganisms (CFU/mL) and by scanning electron microscope (SEM). Also, the surface roughness (Ra) and Vickers microhardness were measured, and color parameters were analyzed using the National Bureau of Standards (NBS) method. Data was analyzed by Wilcoxon and Friedman (microbiological evaluation), paired t-test (color and roughness) and independent t-test (Vickers hardness) (α = 0.05).

**Results:**

A significant reduction (*P* < 0.05) in the number of microorganisms was observed for each species (total microorganisms, *Streptococcus mutans*, and *Candida* spp.), with no significant differences (*P* > 0.05) between hypochlorite and cinnamaldehyde. There was an increase in the roughness and a decrease in the hardness of the test specimens, with no difference between the two disinfectant substances (*P* > 0.05). Both hypochlorite and cinnamaldehyde also caused changes in color, considered as “perceptible” by the NBS classification, but with no significant difference between disinfectant substances (*P* < 0.05), and under the clinically acceptable limit (ΔE ≤ 3.7).

**Conclusion:**

The 27 μg/mL cinnamaldehyde solution was effective against all evaluated microorganisms and caused minor alterations in hardness, surface roughness, and color parameters, with no clinical relevance.

## Background

The increase in life expectancy in developing countries has led to an increase in the number of elderly in the population, and consequently people who need removable complete dentures [1]. Complete dentures users often present denture stomatitis (DS), commonly associated with the presence of *Candida* spp. [2, 3].

These microorganisms inhabit the oral cavity in a commensal way, but local changes or systemic compromises can promote an imbalance, causing candidiasis, which can range from a superficial and localized involvement to a fatal disease, when disseminated in the body of individuals immunocompromised [4].

Appropriate denture hygiene is necessary to eliminate oral bacterial and fungal biofilms and prevent DS. Studies have revealed that several factors influence the choice of the cleaning method used, and that they rely less on evidence-based guidelines than other factors like regional differences, the clinician or patient personal preference, cost, or material availability [5–8]. Some characteristics have been pointed as desirable for an “ideal” denture cleanser: it should have antibiofilm activity, to be nontoxic and compatible with denture materials, should be easy to use, have an acceptable (or no) taste and be cost-effective [9]. Sodium hypochlorite in low concentrations (0.25–0.5%) has demonstrated adequate bactericidal and fungicidal effects and effectiveness in denture disinfection, eliminating biofilm and staining of the denture’s surface, but it has some disadvantages for clinical use [10–13].

In addition to its unpleasant odor and taste, the disadvantages of using sodium hypochlorite, even when used in low concentrations for night-time immersion, are possible color changes, an increase in the roughness of the denture base material, and toxicity [14–16].

Due to its antimicrobial potential, plant products present an opportunity for the discovery of new drugs with antimicrobial activity. Hence, research on natural products in dentistry has increased in recent years, with the promise that new products towards the prevention and treatment of infectious disease will be discovered [17]. One focus is on essential oils, which present antibacterial activity for both Gram-negative and Gram-positive bacteria [18, 19]. The essential oil obtained from the leaves and bark of *Cinnamomum zeylanicum* Blume (cinnamon) is one of the most efficient in inhibiting microbial growth. This is due to the presence of cinnamaldehyde in high concentrations (79.74%) [18–20].

A previous study performed by our group showed that cinnamaldehyde had a fungicidal action against *Candida albicans,* by acting on the fungal cell membrane, interfering with cellular functions, mediated by ergosterol [21]. A clinical study showed the safety of a solution containing *C. zeylanicum* essential oil, which presents cinnamaldehyde as one of the main phytoconstituents, when used as a mouthwash for 15 days [22].

The hypothesis formulated is that cinnamaldehyde is safe and effective for the disinfection of removable complete dentures and, when compared to sodium hypochlorite, it can present equivalent efficacy against fungi and bacteria, with no negative effects of smell and taste, reduced effects on the physical properties of the denture base material, improving the patient’s adherence to the treatment.

The aim of this study was to evaluate the effect of cinnamaldehyde in the disinfection of removable complete dentures and to evaluate its effect on physical and mechanical properties of acrylic resin (Vickers microhardness, color, and surface roughness).

## Methods

### Study design

It was performed a crossover, blind, randomized in situ study, that aimed to investigate the effect of cinnamaldehyde on the disinfection of removable complete dentures, in patients without denture stomatitis. Moreover, we analyzed the effect of these products on the physical properties of the acrylic resin.

In addition, and before conducting the in situ study, an in vitro experiment was carried out to evaluate the effect of cinnamaldehyde against *Candida* multispecies biofilm. The value of the effective concentration of this product, verified in this test, was used for proposing the design of the clinical study. The methodology adopted and the results obtained by the in vitro test are presented in the Materials and Methods.

### Sample selection

The present study was approved by the Research Ethics Committee of the Health Sciences Center of the Federal University of Paraíba (n° 2,303,954), and informed consent was obtained in writing from all patients. Thirty-three volunteers selected from the Dentistry College at Paraíba Federal University, among the ones awaiting replacement of their maxillary complete dentures. For the sample calculation, Microsoft Excel® was used, with parameters of a Confidence Level of 95%; Type I error of 5% two-tailed; Type II error of 20%; Power of 80%; for an Effect Magnitude for a hypothesis of mean difference (0.7); for paired groups. Sample losses were not considered in the sample calculation, due to the high cost for the construction of new dentures, that were provided free of charge to the research subjects after the completion of the study. Fortunately, there was no sample loss during the conduction of the trial.

The inclusion criteria were adult patients of both sexes, any age, good oral and general health, with complete upper edentulism, using a maxillary complete denture constructed with heat-cured acrylic resin, presenting no signs or symptoms of denture stomatitis, but carrying *Candida* spp., with normal salivary flow (0.3–0.5 mL/min), and capable of complying with the experimental protocol. The exclusion criteria included individuals with risk factors for decreased salivary flow, such as diabetes, autoimmune diseases and users of psychiatric drugs, nocturne (during sleeping) maxillary dentures wearers and, current antifungal or antibacterial drugs users or with history of their use 1 month prior to the study.

The volunteers selected were carriers of *Candida* spp., as previously verified by an initial biofilm screening collected from their dentures using a swab. This material was seeded on CHROMagar® Candida plates (Difco®, Le Pont de Claix, France), which were aerophilically incubated at 35 ± 2 °C for 24 h to confirm the presence of the microorganism.

**Fig. 1:**
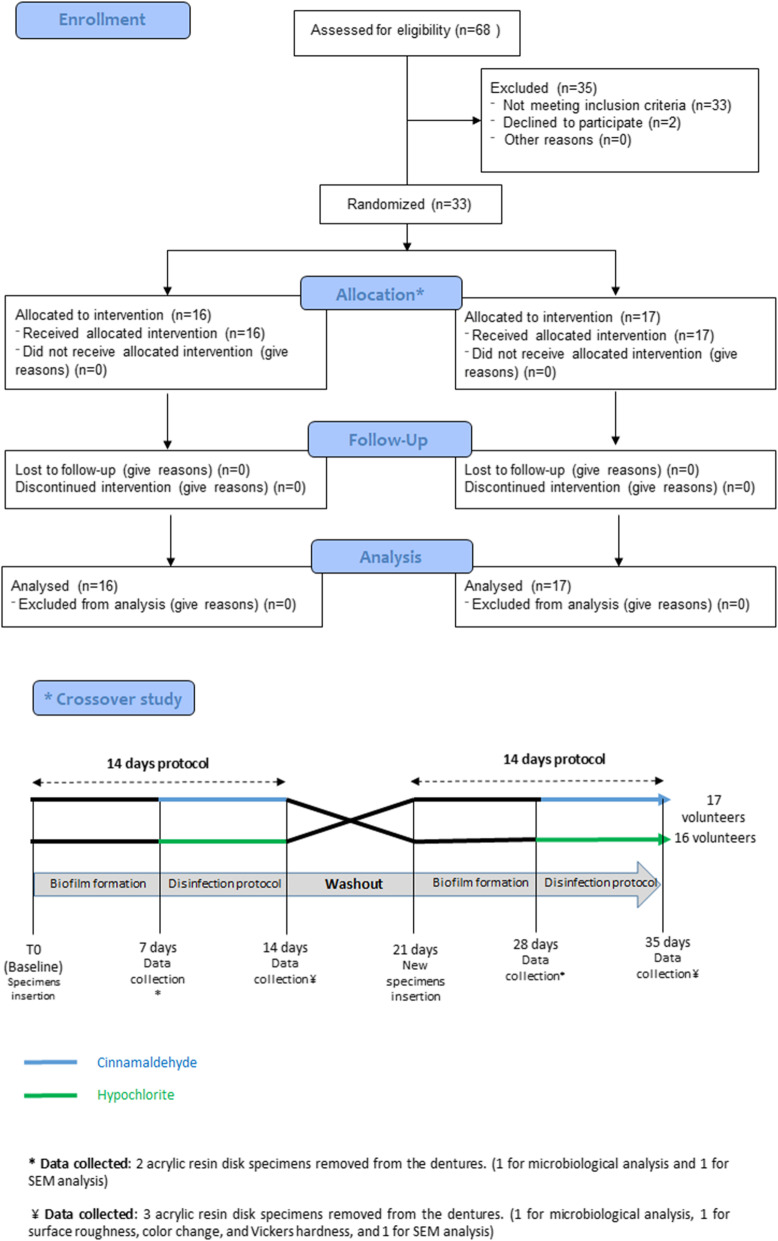
Flowchart of the study participants (Adapted from the Consort Statement)

The in situ phase of the study was researcher blinded with 33 selected volunteers for two 14-day steps, each subdivided into 7 days involving oral biofilm formation on acrylic resin disk specimens inserted in the basal area of the dentures, and 7 days of prostheses immersion in the evaluated products. Thus, each participant was integrated into a simple randomized way as defined by the Random Allocation Software 2.0, for immersing the old dentures containing the specimens into the respective products for disinfection; both the comparison group (hypochlorite 0.5%) and the experimental group (cinnamaldehyde). Figure 1 shows the experimental model adopted.

### In vitro adhesion inhibition test of *Candida* multispecies biofilm

To determinate the better concentration of cinnamaldehyde solution for the in situ study, an in vitro adhesion inhibition test assay was performed. Flat bottom 96-well plates were used to inoculate 200 μL of suspensions of a multispecies (fungal) biofilm (*C. albicans* ATCC 90028; *C. tropicalis* ATCC 750; *C. krusei* ATCC 34135), grown on CSD medium (KASVI®, Kasv Imp, and Dist from Prod Laboratorios LTDA, Curitiba, Brazil), supplemented with 2% sucrose, and adjusted to 2.5 × 10^5^ CFU/mL. After culture, 100 μL of cinnamaldehyde solution (Sigma-Aldrich, São Paulo, Brazil) was transferred onto the multi-species biofilm plates, with concentrations varying from 216 to 6.75 μg/mL (1,634.38–51.07 μM). Sodium hypochlorite was used as a positive control with concentrations between 0.5 and 0.016%. The plates were incubated for 48 h in a microbiological oven at 35 ± 2 °C.

After incubation, the medium was aspirated from the plates, and the unbound cells were removed by washing the wells twice with 200 μL of phosphate buffered saline and drying at room temperature for 45 min. Aqueous crystal violet solution (200 μL, 0.4%) was added to each well and remained in contact with the biofilm for 45 min. After incorporation of the dye, the wells were washed four times with 200 μL of distilled water and immediately bleached for 45 min with 200 μL of 95% ethanol. Finally, 100 μL of the bleached solution was transferred to a new flat-bottom plate and the absorbance measured at 600 nm in a microplate reader.

The cinnamaldehyde and growth control absorbance values ​​were used to calculate the percentage inhibition of biofilm formation. The growth control was considered to be 100% fungal formation. The assays were performed in triplicate. Sterile controls did not receive cell suspension, and growth controls received only culture medium and strains corresponding to the multispecies biofilm.

For this test, were used the concentrations of 216; 108; 54; 27; 13.5 and 6.75 μg/mL of cinnamaldehyde and 0.5; 0.250; 0.125; 0.063; 0.031 and 0.016 μg/mL sodium hypochlorite, which was used as a positive control. It was observed that in the four highest concentrations of cinnamaldehyde there was no significant difference in biofilm reduction, which was around 83% (ANOVA one-way test followed by Tukey’s test). Also, the cinnamaldehyde dose-response curve demonstrated the percentage of biofilm reduction at each concentration tested, being observed a dose-dependent effect and the highest potency at 27 μg/mL, promoting the maximum effect.

### Preparation of acrylic resin disk specimens

**Fig. 2:**
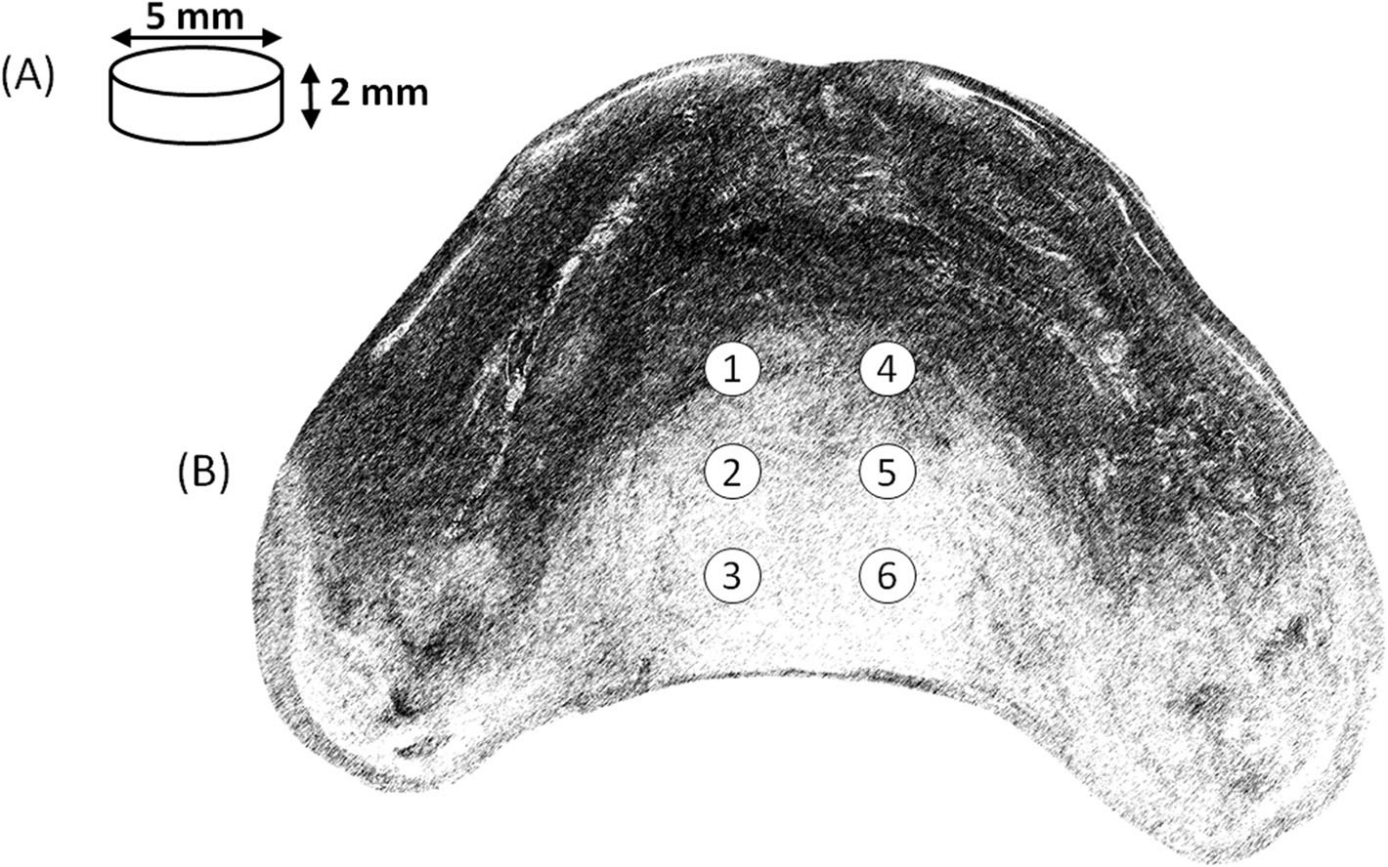
**a** Schematic drawing of the dimensions of the specimens; **b** Schematic view of the insertion sites into the basal area of the dentures. Specimens were randomly removed from the dentures after the 7-days period of biofilm formation (2 specimens) and the remaining 3 specimens were removed after the 7-day period of immersion into the disinfecting solutions. One extra specimen was used if any specimen was lost during the duration of the experiment, during the denture use or hygiene

A microwave heat-cured acrylic resin (VipiWave, Vipi®, São Paulo, Brazil – color pink) was used to prepare the specimens (disks) in the dimensions of 5 × 2 mm. The resin was proportioned, manipulated, included in flasks over a glass plate (to provide a smooth surface), and cured by microwave radiation according to manufacturer’s instructions. Due to the small size of the disks, and the smoothness provided by the glass plate during polymerization, no polishing was performed on the disks. Six test specimens (disks) were fixed with wax into prepared cavities into the intaglio surface of the old prostheses, by using a wheel diamond bur (PM 19, KG Sorensen®, São Paulo, Brazil). Figure 2 presents a schematic drawing of the dimensions of the specimens and the insertion sites into the basal area of the dentures.

### In situ clinical study

The participants were instructed to brush their dentures three times a day (after breakfast, lunch, and dinner) with a specific brush and a neutral liquid soap [23], provided by the researchers, except for the region containing the specimens, which received only the residual foam without mechanical (brush) cleaning [3, 24]. During the first 7 days, the participants used only the mechanical method of hygiene herein described, without immersion of the prosthesis in any product for disinfection. During the following 7 days, the participants immersed the prostheses once daily (for 20 min) in 200 mL of the following solutions: 0.50% sodium hypochlorite [10–12, 14, 23], and 27 μg/mL cinnamaldehyde solution (as determined by the 96-well plate biofilm adhesion inhibition test with *Candida* spp. multispecies).

Participants used one solution for each phase of the study, each for 7 days, according to the randomization process previously explained. After each period, there was a seven-day washout to eliminate residual effects of the previously used solutions, during which the volunteers used only the mechanical method of hygiene with a specific brush and neutral liquid soap [23]. The use of mouthwashes or antimicrobial drugs was not allowed during the study. Written instructions were given to all volunteers concerning hygiene methods and the procedures adopted in the survey.

The prepared solutions were delivered to the research subjects in opaque bottles, containing a volume sufficient for the 7 days immersion protocol (1.5 L). Since the products differ in color, taste, and smell, the bottles were sealed and were not labeled. The researcher responsible for the randomization and production of the solutions was not involved in the clinical study phase, to avoid bias.

After 7 days of biofilm formation, two specimens were randomly removed from the dentures, with an sterilized instruments. One was placed in a sterile Eppendorf and kept in an ice bath until processing as a specimen for culture and counting of colony-forming units (CFU), and one specimen was used for the qualitative analysis by scanning electron microscopy (SEM). The same protocol was used after the next 7-day immersion period in the solutions, when the remaining specimens were removed from the dentures. At this point, one was used for the surface roughness, color change, and Vickers microhardness measurements, and the other 2 were used for CFU and SEM evaluation. The 6th specimen was only used if any specimen was lost during the duration of the experiment, during the denture use or hygiene.

At the end of the first 14-day phase, the niches (test areas) received new specimens (disks) for the start of the second 14-day phase, with the same volunteers, proceeding in the same way previously described for the first phase, with the immersion in the second tested disinfection solution.

The construction of new dentures, which were provided for the volunteers, occurred concurrently with the study. The volunteers always continued to use their old prostheses until the new ones were delivered.

During the study, all patients were monitored (daily phone call) for possible discomfort or undesirable effects due to the use of cinnamaldehyde or hypochlorite, and also answered a satisfaction questionnaire at the end of the study. Questions were: *“Did you feel any discomfort, pain or burning sensation during the use of any of the two cleaning solutions? If so, which one caused it, number 1 or number 2?”* and *“After using both solutions, which was your favorite? Number 1 or 2?”* The methodological design related to the composition of the experimental groups is shown in Fig. 1.

### In situ microbiological evaluation

The specimens collected in the Eppendorf tubes received 1 mL sterile 0.9% NaCl solution and were agitated in the Vortex for 30 s at 7 W. The suspension was serially diluted in saline occurring from 10^− 0^ to 10^− 3^. The dilutions were seeded in Petri dishes containing the culture media: a) Mitis salivarius® (Difco, Le Pont de Claix, France) for determination of *mutans* group streptococci, b) Mueller Hinton® agar (Himedia, Mumbai, India) for determination of total microorganisms, and c) CHROMagar® *Candida* media (Difco, Le Pont de Claix, France) for the determination of *Candida* spp. [3, 23].

Seeding was performed by deposition of 20 μL aliquots of these triplicate dilutions in the Petri dishes with the culture media [25]. The plates containing the CHROMagar® Candida, Mitis salivarius® and Mueller Hinton® media were incubated in an oven at 37 °C for 48 h, with Mitis salivarius® plates maintained under micro anaerobioses atmosphere.

The colony-forming units (CFU) were counted using a stereoscopic microscope and the results expressed in CFU/mL. In addition, the amount of *Candida albicans* and non-albicans *Candida* in relation to total biofilm was calculated.

### Scanning electron microscopy analysis

The scanning electron microscope (SEM) analysis had the purpose of illustrating the surface condition at the 7th day of biofilm formation, and on the 14th day after immersion in both solutions. So, two specimens were randomly selected from the hypochlorite group, and two specimens for the cinnamaldehyde group.

The specimens were fixed with 2.5% glutaraldehyde for 12 h at 4 °C, washed three times in 0.1 M phosphate buffer at 4 °C (pH 7.3) for 10 min each. After fixation, the specimens were dehydrated in 50, 70, 80, 90, 95 and 100% water/ethanol mixtures for 20 min each, and then mounted on a *stub*, and air-dried, (EMITECH K550X). Then, specimens were examined with a scanning electron microscope (ZEISS, model LEO 1430), operated at 5 kV, to characterize the surface of the biofilm formed, focusing on surface morphology [3]. The images were obtained by backscattered electrons and secondary electrons, with magnifications of 2500x and 5000x.

### Color change, surface roughness, and Vickers microhardness evaluation

Color parameters, surface roughness and Vickers microhardness were measured in two moments: before the insertion into the dentures niches, and after the 14th day of the experiment, for both solutions. In this way, the acrylic resin discs were submitted to a complete cycle, covering 7 days of biofilm formation, and 7 days of disinfection with one of the solutions. Due to the fact that Vickers microhardness diamond indenter produces marks on the materials surface, that could possibly affect the surface roughness and color evaluations and be a source of bias, the baseline hardness evaluation was performed in a group of specimens that were not used during the clinical in situ study, and acted only as a control group. From a total of 33 specimens obtained after the experiment, for each solution, a sample of 16 specimens was randomly selected through Microsoft Excel® for the final properties evaluations for each disinfection solution (*n* = 16).

To evaluate the color parameters change (CIE L*a*b) of the specimens after immersion in the solutions, a portable dental spectrophotometer (Vita Easy Shade, Vita ZahnFabrick, Germany) was used. After calibration, the specimens (*n* = 16) were placed on a white surface in order to standardize the color measurement site for all specimens. The spectrophotometer was calibrated according to the calibration standard provided by the manufacturer. Three measurements were made for each specimen, and the mean values were registered for the parameters “L,” “a,” and “b,” where “L” refers to the brightness, “a” means redness to greenness, and “b” yellowness to blueness [26, 27].

Color change (ΔE) was calculated according to the Commission Internationale de l’Eclairage (CIE) L* a* b* with D65 illumination through the following formula [27]:

$$ \Delta {\mathrm{E}}^{\ast }={\left[{\left(\Delta {\mathrm{L}}^{\ast}\right)}^2+{\left(\Delta {\mathrm{a}}^{\ast}\right)}^2+{\left(\Delta {\mathrm{b}}^{\ast}\right)}^2\right]}^{1/2}. $$

In which ΔL, Δa, and Δb refer to the difference among the values of L*, a*, and b* between the initial color measurement and the measurement after the 14-day experiment, for each tested solution. Quantification of ΔE values was performed using the National Bureau of Standards (NBS), with NBS units of color difference (NBS units = ΔE*0.92) [13, 26–28]. A limit of ΔE ≤ 3.7 was considered clinically acceptable [27]..

Surface roughness analysis (Ra) was performed on the non-contact optical profilometer (CCI MP, Taylor Hobson, England). A 0.25 mm cutoff was used with a 50x lens, 0.4 numerical aperture, and × 1 scan speed in *xyz* mode. Three measurements were performed for each specimen (*n* = 16), with the final roughness (μm) obtained as the average of the three points of each specimen [16, 27].

For the Vickers hardness readings, the specimens (*n* = 16) were subjected to three hardness readings in a Shimadzu Microdurometer (HMV Micro Hardness Test, Shimadzu Corporation, Kyoto, Japan), loading 100gf for 30 s [29]. The acrylic resin specimens remained parallel to the microdurometer table and were stable, allowing marking by the Vickers tip. Upon indentation, the operator of the equipment measured the diagonals created by the diamond upon the specimen and the equipment automatically converted the averages into units of Vickers hardness (kg/mm2) with a two-tenths precision scale. After three readings for each specimen, the average was recorded.

### Cost analysis

Only the direct costs involved in the preparation of the disinfections solutions (chemical substances, diluents, consumables, and storage containers) were calculated into the cost analysis. Direct costs of the expenses associated with labor and equipment, and indirect costs (time and resources used by participants to go to the clinic) were not considered. Costs were calculated in United States dollars ($) [30].

### Data analysis

Initially, all data were analyzed to verify the normality of the distribution by Kolmogorov-Smirnov test. The data obtained by microbiological evaluation have show non-parametric distribution, and the results were analyzed by a statistical test for paired non-parametric (Wilcoxon and Friedman) samples.

The results observed to the assessment of acrylic resin properties presented a parametric distribution. The evaluation of surface roughness and calor changes were performed on the same specimens of acrylic resin before and after treatment with the solutions. Thus, for these parameters, the paired *t*-test was considered. The data obtained by Vickers microhardness were analyzed by Independent *t*-test.

All tests were performed considering a level of significance of 5%, using the Microsoft Excel® and BioEstat 5.3 statistical software.

## Results

All participants (*n* = 33) completed the two denture’s disinfection cycles. In Table 1, it was verified a reduction in the number of colony-forming units (CFU/mL) after the use of both substances (*p* < 0.05), between the 7th and 14th day. No significant differences (*p* > 0.05) were verified between hypochlorite and cinnamaldehyde, for all microorganisms. Non-albicans *Candida* colonies were more often found than *Candida albicans* colonies, although this difference was not statistically significant (*p* > 0.05).

**Table 1:** Reduction in colony-forming units (CFU/mL, mean ± SD) before (7-days) and after (14-days) the disinfection protocol with hypochlorite and cinnamaldehyde

Treatment	Total microorganisms (× 10^**4**^)		***S. mutans group*** (× 10^**4**^)		***Candida*** spp. (× 10^**4**^)	
	7 days	14 days	7 days	14 days	7 days	14 days
**Hypochlorite**	344 ± 586 ^a A^	42 ± 67 ^b A^	82 ± 186 ^a A^	7 ± 12 ^b A^	17 ± 62 ^a A^	2 ± 6 ^b A^
**Cinnamaldehyde**	282 ± 862 ^a A^	62 ± 69 ^b A^	69 ± 112 ^a A^	17 ± 35 ^b A^	6 ± 16 ^a A^	1 ± 4 ^b A^

Lowercase letters represent statistically significant differences between the substances (line), before and after utilization of the solution by type of microorganism (Wilcoxon Test)

Uppercase letters represent no difference between the substances (column), (Wilcoxon test)

**Fig. 3:**
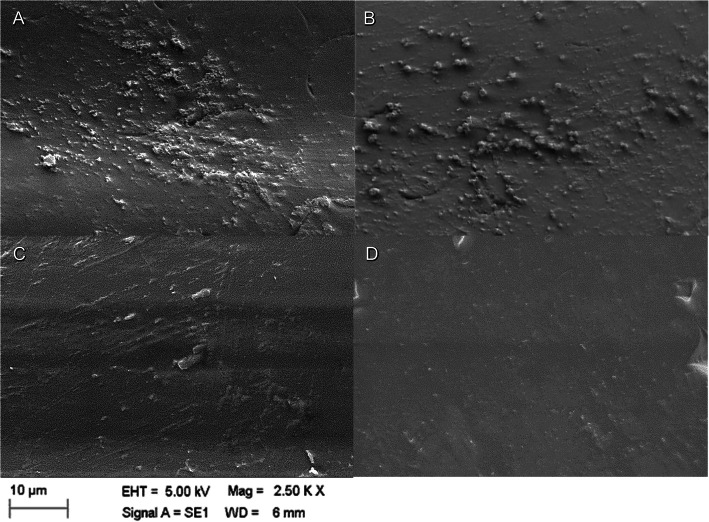
Scanning electron microscopy (SEM) image for qualitative evaluation of colony morphology for each time and solution used. At 7 days: **a** and **b** formation of colonies without using the solutions (original magnification × 2500). At 14 days, **c** solution with hypochlorite; **d** solution with cinnamaldehyde (original magnification × 2500)

The species of *Candida* spp. most often isolated were *C. albicans, C. krusei, C. tropicalis*, and *C. glabrata*, with *C. krusei* predominance in the first 7 days of biofilm formation on the acrylic resin. In the qualitative analysis performed by scanning electron microscopy (SEM), a considerable reduction in the number of colonies between the biofilm formation period (7th day), and product use (14th day) was observed, for both hypochlorite and cinnamaldehyde (Fig. 3).

After the use of the solutions to disinfect the dentures, there was an increase in roughness and a decrease in the hardness of the specimens for both products (Table 2), with significant difference within the pairing (*p* < 0.05), but not between the substances (*p* > 0.05).

**Table 2:** Surface roughness (Ra) and Vickers microhardness (mean ± SD) of acrylic resin specimens at baseline and after the 14-day experiment

Treatment	Surface roughness Ra (μm, mean ± SD)		Vickers microhardness VHN (kg/mm^**2,**^ mean ± SD)	
	Baseline	14th day	Baseline	14th day
**Hypochlorite**	0.047 ± 0.010 ^a A^	0.059 ± 0.019 ^b A^	20.80 ± 0.95 ^a A^	20.08 ± 0.46 ^b A^
**Cinnamaldehyde**	0.050 ± 0.015 ^a A^	0.056 ± 0.014 ^b A^	20.80 ± 0.95 ^a A^	19.98 ± 0.48 ^b A^

Lowercase letters represent statistically significant differences between the substances (line), before and after the use of the solution (paired t-test)

Uppercase letters represent no difference between the substances (column), (Independent t-test)

When analyzing the color change parameters of the acrylic resin (Table 3), it was observed that both hypochlorite and cinnamaldehyde promoted changes considered perceptible by the NBS classification, with no significant difference between the products (*p* > 0.05). However, ΔE values were considered clinically acceptable for both substances (ΔE ≤ 3.7).

**Table 3:** Color change (Delta L, Delta a, Delta b, ΔE, and NBS units) of acrylic resin specimens after the 14-day experiment (mean ± SD)

Treatment	Delta L	Delta a	Delta b	ΔE	NBS Units	NBS Color difference
**Hypochlorite**	−0.78 ± 0.70 ^a^	−1.07 ± 0.91 ^a^	−0.63 ± 1.19 ^a^	1.97 ± 0.95 ^a^	1.81	Perceptible
**Cinnamaldehyde**	−0.60 ± 0.94 ^a^	−1.07 ± 1.55 ^a^	−0.57 ± 1.79 ^a^	2.54 ± 1.25 ^a^	2.34	Perceptible

No difference between the substances (column) (paired t-test)

Cost analysis indicated that $ 0.52 was required to prepare 10 L of the cinnamaldehyde solution (27 μg/mL), while $ 1.30 was necessary for the same amount of 0.5% sodium hypochlorite.

All 33 patients responded to the satisfaction questionnaire at the end of the study, and 93% of the patients who used cinnamaldehyde and 97% of those who used hypochlorite presented no discomfort after the dentures disinfection process. Most of the patients (61%) preferred the solution containing cinnamaldehyde.

## Discussion

Complete dentures constructed with acrylic resin usually present wear and an increase in surface roughness over time. This is usually associated with mechanical brushing, which is effective in removing surface biofilm. However, dentures present microscopic defects, such as superficial pores and grooves, which may be inaccessible to the brush, and harbor microorganisms that are only removed by chemical disinfection. Sodium hypochlorite, even in small concentrations is one of the most effective solutions used for dentures immersion protocols [7, 31].

Sodium hypochlorite (0.5%) presented antimicrobial activity against all of the microorganisms tested, including *S. mutans* spp. and *Candida* spp., which are frequently found in patients with denture stomatitis, which confirms its efficacy in the control of denture biofilm [3, 11, 12, 23]. It promotes a substantial reduction in viable cells of both *Candida albicans* and non-albicans *Candida* [10], but in concentrations higher than 0.05%, it presents cytotoxicity to fibroblast cells [15, 32]. Its mechanism of antimicrobial action involves physicochemical effects, altering the integrity of the cytoplasmic membrane, causing irreversible enzymatic inhibition and biosynthetic changes to the cellular metabolism, and resulting in cell death [33].

In this study, the concentration of the cinnamaldehyde solution used was 27 μg/mL, which is considered to have a very strong activity [34]. The cinnamaldehyde effect was similar to hypochlorite, presenting antimicrobial activity against all of the tested microorganisms. Other studies have shown that cinnamaldehyde presents fungicidal activity, starting from a 40 μg/mL concentration, causing changes to *Candida* spp. membrane and interior [35]. A 312 μg/mL concentration is effective against already established biofilms [18, 36]. Inferior concentrations, such as 156 μg/mL or 234 μg/mL were able to reduce bacterial counts during biofilm formation [18] and exhibit excellent antibacterial activity against *S. mutans*, *S. sobrinus* and *Staphylococcus aureus* [20].

Cinnamaldehyde dissolves the lipids of the cell membrane and mitochondria, making them permeable, and leading to cellular leakage [37–39]. Gram-negative bacteria are less sensitive than Gram-positive bacteria [37].

The 27 μg/mL cinnamaldehyde concentration used in this investigation was previously determined for the study by an in vitro adhesion inhibition test against *Candida* spp. multispecies, promoting a significative reduction (82%) of *Candida* spp. colonies, results similar to the clinical study (77% of reduction). The 0.5% sodium hypochlorite concentration promoted similar results, with 82% in vitro reduction of *Candida* spp. colonies, and 88% in the clinical study. SEM images showed the colonies’ reductions after disinfection. These results suggest the superiority of the cinnamaldehyde pharmacological effect, promoting an efficacy similar to sodium hypochlorite, but in reduced concentrations.

Denture stomatitis is not only the result of the presence of *Candida* spp., but a result of multiple species biofilms [3, 40]. *C. albicans, S. aureus,* and *S. mutans* frequently colonize the oral mucosa of denture users. Biofilms are frequently found in patients with denture stomatitis, presenting less colonization of the complete dentures than of the oral mucosa [40]. Since *S. mutans* appear in the initial stages of biofilm development and is commonly found on acrylic denture surfaces, it collaborates with *Candida* spp. in the etiopathogenesis of denture stomatitis, contributing to yeast adhesion [3, 40]. Thus, for a product to be considered effective for disinfection of acrylic dentures, and contribute to the prevention and treatment of denture stomatitis, it must act against these microorganisms. This occurs with cinnamaldehyde and hypochlorite, and without significant differences.

It is worth mentioning that the *Candida* spp. species found in this study, as well as the predominance of *C. krusei* found in the first 7 days of biofilm formation (on the acrylic resin), have already been reported in another in situ study [24].

When analyzing the effect of the disinfection solutions on the properties of the acrylic resin, there was a significant reduction in the Vickers hardness (from the baseline) for both substances. During the chemical disinfection process, water sorption may eventually cause irreversible damage to the material through the formation of micro-fissures due to repeating sorption/desorption cycles, all this contributing to the reduction of hardness [41]. A small reduction in the microhardness of up to 2.57 VHN for the acrylic resin is equivalent to the reduction promoted by artificial saliva [22]. Although this small but significant reduction occurred, it cannot be considered clinically relevant, because there are no hardness thresholds reported in the literature, and they are not expected to promote clinically significant damage to the surface [42, 43]. Thus, to complement the mechanical cleaning of the dentures, the implementation of a daily immersion protocol with either of these chemical disinfection solutions would not significantly reduce the hardness of the denture base [43].

There was also a significant increase in surface roughness (from the baseline) through the use of these solutions for disinfecting the dentures, with no significant difference between them. It is important to note that similar baseline roughness values have already been reported in the literature, demonstrating an adequate standardization of the sample’s surface [44]. It has been shown that the use of a mechanical method (brushing) without dentifrice, combined with immersion in solutions for disinfection, especially sodium hypochlorite, does not cause a clinically relevant increase in the roughness of the resin [45] and small increases in roughness (up to 0.04 μm) are compatible with changes caused by deionized water [43].

Roughness average (Ra) values up to 0.2 μm are considered clinically acceptable and they difficult biofilm formation and microbial adhesion to the denture surface, and, from this point of view, none of the tested solutions did promote clinically relevant alterations to the surface roughness of the acrylic resin [16, 27, 42, 43]. *Candida albicans* require larger surface depressions and scratches than bacteria (> 1 μm) to increase retention [46]. It is worth mentioning that the presence of microorganisms in the biofilm formation and maturation process can contribute, in isolation, to the increase in surface roughness of the acrylic resin by up to 0.27 μm [24].

Considering the color change parameters, both solutions caused color changes that were classified as perceptible by the NBS scale. However, sodium hypochlorite ΔE values (ΔE 1.97) are similar to other reported in other studies [16, 47], and the ΔE values promoted by both solutions may not clinically relevant because they are under the clinically acceptable limit (ΔE ≤ 3.7) [13, 26–28]. The values of ΔE for the acrylic resins of the denture base may increase with exposure time [26], and be a cause of dentures color change through repeating sorption/desorption cycles, and result in the formation of micro-fissures and different zones with different optical properties [44, 47].

Finally, both solutions had a similar antimicrobial effect, did not promote significant changes in the color, microhardness, and roughness of the acrylic resin, for the short-term disinfection regime evaluated, showing that they are good options for acrylic complete dentures chemical disinfection. Furthermore, cinnamaldehyde is more biocompatible, a low toxicity substance, and is an odourant molecule, which is an essential component of cinnamon oil and causes the characteristic smell [48, 49]. This may explain the 61% preference for the cinnamaldehyde solution demonstrated by the patients in the satisfaction questionnaire. Users reported low discomfort rates with both solutions (93% for cinnamaldehyde and 97% for sodium hypochlorite), and cinnamaldehyde solution cost were 40% of the cost estimated to prepare an equivalent volume of 0.5% hypochlorite solution, which is another advantage.

Considering the “ideal” denture cleanser desirable characteristics pointed early: adequate antibiofilm activity, absence of toxicity, compatibility with denture materials, easiness to use, acceptable taste, and cost-effectiveness [9], it seems valid to say that cinnamaldehyde is a good candidate for this position. So, considering the limitations of this study and from a clinical perspective, cinnamaldehyde seems a promising substance for further studies focusing on the chemical disinfection of acrylic complete dentures.

## Conclusion

The 27 μg/mL cinnamaldehyde solution tested was effective against all tested microorganisms, including *Candida* spp. and *S. mutans* spp., promoting clinically non-relevant alterations to the Vickers microhardness, surface roughness, and color of the acrylic resin, comparable to 0.5% sodium hypochlorite. Further studies are suggested to evaluate the association with a mechanical method for cleaning and disinfection of complete dentures.

## Data Availability

The datasets used and/or analyzed during the current study are available from the corresponding author upon reasonable request.

## Abbreviations

NBS: National Bureau of Standards
Ra: Roughness analysis
DS: Denture stomatitis
CFU: Colony-forming units
SEM: Scanning electron microscopy
